# Effects of N-acetylcysteine on the inflammatory response and bacterial translocation in a model of intestinal obstruction and ischemia in rats

**DOI:** 10.1590/acb371204

**Published:** 2023-01-13

**Authors:** Rafael Izar Domingues da Costa, Joao Marcos da Silva Fischer, Roberto Rasslan, Marcia Kiyomi Koike, Edvaldo Massazo Utiyama, Edna Frasson de Souza Montero

**Affiliations:** 1 PhD. Universidade de São Paulo – Division of General Surgery and Trauma – Department of Surgery – School of Medicine – São Paulo (SP), and Universidade Federal de São Carlos – São Carlos (SP), Brazil.; Universidade Federal de São Carlos, São Carlos, SP, Brazil; 2 MD. Universidade Federal de São Carlos – São Carlos (SP), Brazil.; 3 PhD. Universidade de São Paulo – Division of General Surgery and Trauma – Department of Surgery – School of Medicine – São Paulo (SP), Brazil.; 4 PhD. Universidade de São Paulo – Department of Clinical Medicine – Laboratory of Emergency Medicine – School of Medicine – São Paulo (SP), Brazil. And Instituto de Assistência Médica do Servidor Publico Estadual (IAMSPE) - Pós-Graduação em Ciencias da Saúde, São Paulo (SP), Brazil.; Instituto de Assistência Médica do Servidor Publico Estadual, Pós-Graduação em Ciencias da Saúde, São Paulo, SP, Brazil; 5 PhD, full professor. Universidade de São Paulo – Division of General Surgery and Trauma – Department of Surgery – School of Medicine – São Paulo (SP), Brazil.; 6 PhD, associate professor. Universidade de São Paulo – Division of General Surgery and Trauma – Department of Surgery – School of Medicine – São Paulo (SP), Brazil.

**Keywords:** Acetylcysteine, Bacterial Translocation, Mesenteric Ischemia, Intestinal Obstruction

## Abstract

**Purpose:**

To evaluate effect of N-acetylcysteine (NAC) associated with Ringer lactate or hypertonic saline in inflammation and bacterial translocation on experimental intestinal obstruction (IO).

**Methods:**

Wistar rats was subjected to IO. Six or 24 hours after, rats were subjected to enterectomy and fluid resuscitation: IO, RL (subjected to the same procedures but with fluid resuscitation using Ringer’s lactate solution); RLNAC (added NAC to Ringer’s solution); and HSNAC (surgical procedure + fluid reposition with 7.5% hypertonic saline and NAC). After 24 h, tissues were collected to cytokines, bacterial translocation, and histological assessments.

**Results:**

In kidney, interleukin-1beta (IL-1beta) was lower in the groups with fluid resuscitation compared to IO group. The RLNAC showed lower levels compared to the RL. Interleukin-6 (IL-6), interleukin-10 (IL-10), tumor necrosis factor-alpha (TNF-alpha), and (*IFN-gamma*) were lower in the treatment groups than in IO. In lung, IL-1beta and IL-6 were lower in RLNAC compared to IO. IL-10 was lower in RL, RLNAC and HSNAC compared to IO. TNF-alpha was higher in HSNAC compared to both RL and RLNAC. Bacterial translocation was observed in all animals of IO group. In kidneys, inflammation and congestion degrees were lower in HSNAC compared to RL. In lungs, inflammation levels were higher in RLNAC compared with the sham group.

**Conclusions:**

The data indicates that NAC associated with RL can promote a decrease in the inflammatory process in the kidneys and lungs in rats, following intestinal obstruction and ischemia in rats.

## Introduction

Mechanical bowel obstruction is an urgency with elevated morbidity and mortality. In the USA, 30,000 deaths occur every year as a result of this pathology^1^. Hernias, tumors, and adhesions are the most common causes, being the third one responsible for 60% of all cases^2^.

After occlusion, arterial compression or increased intraluminal pressure may cause congestion, favoring bacterial proliferation^3^. This complex obstruction leads to systemic inflammatory response syndrome, mainly due to bacterial translocation, a process in which microbes reach the bloodstream via the lymphatic tissues. One of the main consequences is injury to the microcirculation, leading to vasodilation^4^.

Previous research has reported that, in order to reestablish homeostasis, it is necessary to reinstitute intravascular volume. Since great volumes of crystalloid solutions are required to do so, other fluids that demand less volume, such as hypertonic saline, were tested, with conflicting outcomes. On one hand, there were promising results of reduced complications related to edema. On the other one, positive immunoregulatory role in septic patients^5^^-^ 7.

More recent attention has focused on N-acetylcysteine (NAC). It was shown to be capable of reducing and modulating inflammatory mediators, as well as reactive oxygen species^8^. Its anti-inflammatory and antioxidative properties proved to be efficient in septic patients, ameliorating oxygenation and lung compliance^9^.

Experimental models of sepsis cited by Prauchner claimed that NAC had the capability of diminishing inflammatory markers, preventing the decrease of mean arterial pressure, and downregulating the expression of NF-kB in lungs. Consequently, it makes the inflammatory response smaller at this site^10^.

The present investigation aimed to evaluate the effect of NAC associated with Ringer lactate or hypertonic saline in the inflammatory response, as well as bacterial translocation on experimental intestinal obstruction and ischemia.

## Methods

This study was conducted in accordance with the International Guiding Principles for Biomedical Research Involving Animals, being approved by the Research Committee of the Department of Surgery and the Ethics Committee in Use of Animals of the School Medicine of the Universidade de São Paulo (number 0429/13).

Adult male Wistar rats weighting 250-300 g were kept in cages, where humidity and temperature were controlled. There was free access to food and water. The rats were exposed to artificial light, in 12-hour light and dark cycles. They were randomly assigned to four groups, as follows: IO (submitted to intestinal obstruction and ischemia followed by enterectomy with intestinal anastomosis, without fluid resuscitation); RL (subjected to the same procedures but with fluid resuscitation using Ringer’s lactate solution); RLNAC (added NAC to Ringer’s solution); and HSNAC (surgical procedure + fluid reposition with 7.5% hypertonic saline and NAC). There were 10 animals in each group. A sham group was created and had five animals. They were submitted to anesthesia, blood, and organs collection, and were euthanized afterwards.

### Procedure

Animals were anesthetized with ketamine (60 mg/kg) and xylazine (10 mg/kg), intramuscularly. A median laparotomy incision was performed with ligature of the small bowel loop 1.5 cm proximal to the ileocecal valve. The mesenteric vessels, segment between 7 and 10 cm up to the bowel obstruction point, were also ligated. The abdominal wall was closed, and the animals were sent for a 6-hour recovery with free access to water.

Afterwards, the animals were anesthetized again. Then, the obstructed segment was excised, with end-to-end anastomosis of the ileum using polypropylene 6. The incision was closed, and the animals received fluid resuscitation, according to the randomized group, as follows: sham and IO: none; RL: Ringer’s lactate solution 32 mL/kg in 10 minutes; RLNAC: Ringer’s lactate solution 32 mL/kg + NAC 150 mg/kg in 10 minutes; and HSNAC: 7.5% hypertonic saline 4 mL/kg + NAC 150 mg/kg in 10 minutes.

Free access to food and water was offered for 24 hours. Following the protocol, after blood, kidney, lung, liver, spleen, and mesenteric lymph nodes samples were collected, the animals were euthanized by exsanguination.

### Arterial blood gas analysis and lactate, leucometry, and granulocytes

The analyses were performed twice: first (T 6h), at the reoperation, by sampling the right carotid artery, and second (T 24h), at the euthanasia, by direct aortic sampling. In each moment, 0.1 mL of blood was collected. It was analyzed in the automatic device Radiometer ABL 555 (Radiometer Medical, Copenhagen, Denmark).

Leucometry and granulocytes were evaluated in 0.01 mL of blood from the tail’s tip. This analysis was carried out in the automated machine Mindray BC-2800Vet (Shenzhen Mindray BioMedical Electronics Co., Ltd. China).

### IL-1beta, IL-6, IL-10, TNF-alpha and IFN-gamma in lung and kidney

Tissue from lungs and kidneys were homogenized in a solution containing: 10 mM of Tris-HCl, 1 mM of EDTA, 1% Triton X-100 and 2 mM of phenylmethanesulfonyl fluoride (PMSF – Sigma). One μL/mL of protease inhibitor (Sigma) was added to each sample, followed by 10 minutes of 11,000 g centrifugation at 4 °C. The supernatants were subjected to sandwich enzyme-linked immunosorbent assay (ELISA) (Molecular Devices Spectramax – LLC, CA, United States of America) using R & D Systems (Minneapolis, MN, United States of America) reagents for detection of the levels of the mentioned cytokines, following the manufacturer instructions. The samples were quantified by serial dilutions compared with standard purified recombinant cytokine curves. The values were expressed as means of individual samples in pg/mL.

### Bacterial translocation by microbiological culture of mesenteric lymph nodes, lung, liver, and spleen

Liver, spleen, lungs, and mesenteric lymph nodes tissues were obtained prior to euthanasia and homogenized in 0.9% sterile saline. Samples were put into Mac Conkey (MC) (Difco) culture medium and incubated for 24-48 hours at 27 °C. *Escherichia coli* colonies were identified and manually counted, being expressed in colon forming units (CFU) per gram of tissue.

Bacterial translocation was characterized by a positive bacterial culture of mesenteric lymph nodes and the presence of bacteria in lung, liver, or spleen samples.

### Histological analysis of the kidneys and lungs

Tissue fragments were fixed using 10% buffered formalin up to 48 hours and embedded in paraffin. Histological 4 to 5-μm cuts were made. They were put into glass blades and colored with hematoxylin and eosin. They were digitalized and analyzed by a pathologist in two different moments in a blind mode, using Panoramic Viewer version 4.5 (3DHistech, Budapest, Hungary). Semi-quantitative analysis was performed. Scores were given according to the intensity of alterations:

- 0: absent;
- 1: discrete;
- 2: moderate;
- 3: intense;
- 4: very intense.

The lung parameters were evaluated as follows: edema/septal thickening, inflammatory infiltrate, congestion, and alveolar rupture. In the kidney samples, inflammatory infiltrate, congestion, and edema were assessed.

### Statistical analysis

Data were analyzed with GraphPad Prism version 9.0 (GraphPad Software, United States of America), expressed in mean ± standard error of mean or median (interquartile range). Groups were compared by one-way analysis of variance (ANOVA) or Kruskal-Wallis’ test, according to normality pattern, being complemented by post-hoc Student-Newman-Keuls’ or Dunn’ tests, respectively. P-value of 0.05 was adopted for significance level.

## Results

### Arterial blood gas and lactate, leucometry, and granulocytes

The parameters evaluated were similar in all groups 6 hours post-injury. Therefore, they were grouped as a single control value. As shown in Table 1, the values of the 24-h-post-enterectomy samples were compared.

**Table 1:** Arterial blood parameters and lactate measured in 6 hours after intestinal obstructionwas similar among groups and 24h post-enterectomy.

	6 hours	24 hours			
		IO	RL	RLNAC	HSNAC
pH	7.3 (7.3–7.4)	7.4 (7.4–7.5)*	7.3 (7.3–7.4)^¶^	7.3 (7.3–7.4)^¶^	7.3 (7.3–7.4)^¶^
Bicarbonate	19.5 (17–23)	24.9 (24–27)*	20.9 (16–24)	25.5 (23–26)*	21.9 (20–27)
Base excess	-4.6 (-7.2–2.1)	1.6 (1.1–2.1)*	-3.5 (-8–1.8)^¶^	-0.2 (-1.6–0.9)*	-4.3 (-6.2–0.5)
Lactate	0.8 (0.6–0.9)	1.6 (1.2–1.7)	2.1 (1.4–3.7)*	1.6 (1.4–2.1)*	1.7 (1.1–3.9)*

IO: intestinal obstruction; RL: subjected to the same procedures but with fluid resuscitation using Ringer’s lactate solution; RLNAC: added N-acetylcysteine to Ringer’s solution; HSNAC: surgical procedure + fluid reposition with 7.5% hypertonic saline and N-acetylcysteine;

* p < 0.05 vs. 6 hours;

¶ p < 0.05 vs. IO.

The pH levels were higher in IO, when compared to those in sham group. The groups RL, RLNAC and HSNAC showed lower values than IO group. Bicarbonate levels were comparable among groups. Base excess analysis showed difference between: IO and control, RLNAC and control, and IO and RL groups. Finally, considering RL, RLNAC and HSNAC groups, lactate values were higher than the ones presented in the control group.

When it comes to leucometry, there was no difference regarding different moments and groups, as seen in Table 2. In granulocyte count, there was a significant increase in all groups, when the samples were compared before and 6 hours after intestinal obstruction. There was also an increase in the control values 6 hours post-obstruction, as well as in RL, RLNAC and HSNAC.

**Table 2:** Leucometry measured before the procedures, in 6 hours after intestinal obstruction and 24 hours post-enterectomy.

	Control	6 hours	24 hours			
			IO	RL	RLNAC	HSNAC
Leukocytes	13.7 (12–16)	15.7 (12–18)	13.5 (13–18)	14.7 (12–18)	15.8 (12–24)	14.1 (11–21)
Granulocytes	4.4 (3.9–5.5)	10 (8.5–14)*	6.9 (5.7–11)	7.9 (6.2–10)*	6.6 (5–11)*	7.6 (5.4–13)*

IO: intestinal obstruction; RL: subjected to the same procedures but with fluid resuscitation using Ringer’s lactate solution; RLNAC: added N-acetylcysteine to Ringer’s solution; HSNAC: surgical procedure + fluid reposition with 7.5% hypertonic saline and N-acetylcysteine;

* p < 0.05 vs. control.

### IL-1beta, IL-6, IL-10, TNF-alpha and IFN-gamma in lung and kidney

**Figure 1:**
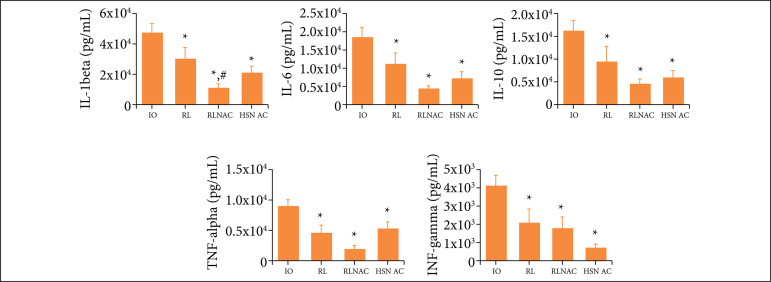
IL-1beta, IL-6, IL-10, TNF-alpha and IFN-gamma in kidney of rats submitted to distinct treatmentin a model of IO and treatment of RL, RLNAC or HSNAC, and 24 hours post-enterectomy.

IO: intestinal obstruction; RL: subjected to the same procedures but with fluid resuscitation using Ringer’s lactate solution; RLNAC: added N-acetylcysteine to Ringer’s solution; HSNAC: surgical procedure + fluid reposition with 7.5% hypertonic saline and N-acetylcysteine; IL-1beta: interleukin-1beta; IL-6: interleukin-6; IL-10: interleukin-10; TNF-alpha: tumor necrosis factor-alpha; IFN-gamma: interferon-gamma; *p < 0.05 vs. IO; #p<0.05 vs. RL; $p<0.05 vs. RLNAC.

In kidney tissue, IL-1beta levels were lower in the three groups that received fluid resuscitation, when compared to the levels of the IO group. The RLNAC group also showed lower levels, when compared to the ones of the RL group. IL-6, IL-10, TNF-alpha and IFN-gamma levels were also lower in the treatment groups than in the IO group (Fig. 1).

**Figure 2:**
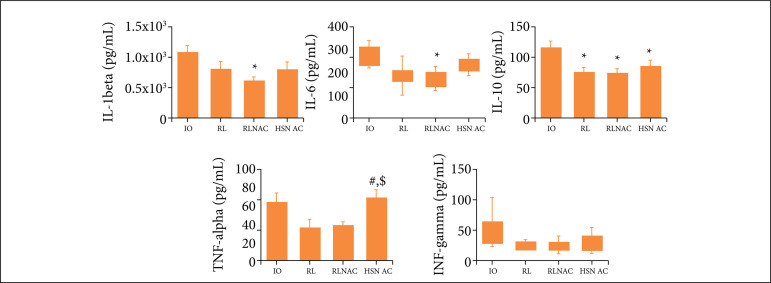
IL-1beta, IL-6, IL-10, TNF-alpha and IFN-gamma in lung of rats submitted to distinct treatmentin a model of IO and treatment of RL, RLNAC or HSNAC, and 24 hours post-enterectomy.

IO: intestinal obstruction; RL: subjected to the same procedures but with fluid resuscitation using Ringer’s lactate solution; RLNAC: added N-acetylcysteine to Ringer’s solution; HSNAC: surgical procedure + fluid reposition with 7.5% hypertonic saline and N-acetylcysteine; IL-1beta: interleukin-1beta; IL-6: interleukin-6; IL-10: interleukin-10; TNF-alpha: tumor necrosis factor-alpha; IFN-gamma: interferon-gamma; *p < 0.05 vs. IO; ^#^p<0.05 vs. RL; ^$^p<0.05 vs. RLNAC.

In lung tissue, IL-1beta and IL-6 were lower with statistical significance in RLNAC, when compared to those in IO. IL-10 was lower in RL, RLNAC and HSNAC, when compared to the ones in IO. No difference among the levels was mapped in the other groups. TNF-alpha was higher in HSNAC when compared to the values of both RL and RLNAC. IFN-gamma analysis showed no difference among groups, as presented in Fig. 2.

### Bacterial translocation

When it comes to bacterial translocation, all animals in IO presented it. Nevertheless, no difference could be observed among groups regarding the number of positive cultures of mesenteric lymph nodes and other tissue, as shown in Table 3.

**Table 3:** Bacterial translocation by positive culture of mesenteric lymph nodes in animals submitted to distinct treatment in a model of intestinal obstruction, and 24 hour post-enterectomy.

Positive culture	Control	IO	RL	RLNAC	HSNAC
Yes	0	8	8	7	8
No	5	0	1	3	1

IO: intestinal obstruction; RL: subjected to the same procedures but with fluid resuscitation using Ringer’s lactate solution; RLNAC: added N-acetylcysteine to Ringer’s solution; HSNAC: surgical procedure + fluid reposition with 7.5% hypertonic saline and N-acetylcysteine

### Histological analysis of the kidneys and the lungs

In kidneys, inflammation and congestion degrees were lower in HSNAC, when they were compared to those in RL, with no difference among other groups. Edema was similar among groups. In lungs, inflammation levels were higher in RLNAC, when compared with the ones presented in the sham group. Moreover, SHNAC showed lower degrees of inflammation than both RL and RLNAC. The congestion degree was also lower in SHNAC than in RLNAC. There was no difference in edema and alveolar rupture levels (Table 4).

**Table 4:** Kidney and lung histological evaluation in animals submitted to distinct treatmentin a model of intestinal obstruction, and 24 hours post-enterectomy^@^.

Histological criteria	IO	RL	RLNAC	HSNAC
Kidney inflammatory infiltratre	1 (1–1)	1.5 (1–2)*	1 (1–1.2)*	1 (0.8–1)
Kidney congestion	1 (1–1.5)	2 (1.8–2)*	1 (1–2)	1 (1–1.3)#
Kidney edema	1 (0.5–1.5)	1 (1–2)	2 (1–2)	1 (1–2)
Pulmonar edema/septal thickening	1 (1–2)	1 (1–2)	2 (0–2)	0 (0–1)
Pulmonar inflammatory infiltrate	1 (1–2)	2.5 (2–3)*	3 (3–4)*	1 (1–1.3) $
Pulmonar congestion	1 (1–2)	2 (1–2)	2 (2–2.3)*	1 (1–1) ^$^
Pulmonar alveolar rupture	0 (0–0.5)	1 (0–1.3)	1.5 (0.8–2)*	0 (0–1)

IO: intestinal obstruction; RL: subjected to the same procedures but with fluid resuscitation using Ringer’s lactate solution; RLNAC: added N-acetylcysteine to Ringer’s solution; HSNAC: surgical procedure + fluid reposition with 7.5% hypertonic saline and N-acetylcysteine;

@ values expressed in median (interquartile range)

* p < 0.05 vs. IO;

# p < 0.05 vs. RL;

$ p < 0.05 vs. RLNAC.

## Discussion

According to our findings, resuscitation with both fluids attenuated inflammation in renal and pulmonary parenchyma, especially when associated with NAC. Additionally, no changes in morphology could be identified. The use of NAC and Ringer lactate promoted a decrease in IL-1beta and IL-6 in the lung tissues. These results suggest that it may reduce inflammation caused by intestinal obstruction. These findings are corroborated by literature. Previous studies, using a model of hemorrhagic shock, pointed out lung injury attenuation in animals that were treated with NAC and Ringer lactate^11^, as well as in those treated only with NAC^12^.

TNF-alpha levels were slightly lower in RL and RLNAC groups, when compared to those in IO. Notwithstanding, HSNAC showed a significant increase, as its levels were compared with those of the other two intervention groups. A previous study by Yang *et al.* 13 showed reduction in the levels of TNF-alpha in animals submitted to 1 hour of intestinal ischemia and treated with HS. This difference could be a result of the difference regarding time of ischemia (1 vs. 6 hours)^14^.

Another investigation showed an increase in inflammatory markers and histological lesion scores when the infusion of hypertonic saline was made 90 minutes after the induction of endotoxemia, when compared to that performed 15 minutes later^15^, including levels of IL-10. In our study, the levels of IL-10 and INF-gamma showed no significance between therapeutic groups, with all of them being significant lower than IO group, perhaps again influenced by the time of resuscitation and sampling.

In renal parenchyma, cytokine levels were higher in the group without treatment. RLNAC had significant lower levels, especially IL-1beta. In the same vein, Ergin *et al*.^16^ found reduced levels of acute kidney injury biomarkers (NGAL and L-FABP) in a model of endotoxemia. Another study presented evidence of lower cytokine levels, suggesting that elevation of glutathione levels protects the kidneys in the same kind of model^17^.

Time intervals for the interventions were defined in a preliminary study, in order to make bacterial translocation and inflammation response studies possible^14^. Lymph node culture was used to assess bacterial translocation. All cultures were negative in the sham group and positive in IO group, proving it to be a trustworthy model for this analysis.

All intervention groups demonstrated reduction of translocation, with no significant differences among them. Timing of intestinal ischemia and obstruction may explain this, since differences in the time of fluid resuscitation were not analyzed in our preliminary study^14^. Consequently, further investigations are necessary.

Edema and alveolar rupture were similar in the intervention groups. Inflammation and congestion were significantly lower, when comparing SHNAC to RLNAC. This is probably related to lower volumes of fluids. In an experimental study, Kolomaznik *et al*.^18^ showed benefits of using NAC in rat model of endotoxemia. It was demonstrated by the reduction of inflammatory markers and oxidative stress.

Arterial blood gas analysis presented a great variety of results, which compromised the analysis. A possible explanation could be the moment of sampling. The first sample was carried out when the animals were anesthetized for the enterectomy, and the second one after the animals were anesthetized for being sacrificed, and not earlier after the resuscitation. Shih *et al*.^19^ demonstrated an improvement in the values of blood gas analysis, when sampling occurred in two moments: 3 and 9 hours after lesion. This could serve as a guidance for further studies, aiming to determine the best moment to obtain arterial sampling after induction of lesions, and mainly after treatment.

There were limitations regarding the evaluation of the positive culture numbers among groups. This is due to the fact that there was not clearly uniformity in the results among the groups with 6 hours of injury, and there was high mortality (60%) in 24-hour regimens in our prior study^14^. A possible solution for this is reducing the time prior to enterectomy and anastomosis, in order to make mortality rates acceptable while inducing the desired changes more homogeneously.

## Conclusions

This research was designed to evaluate the effect of NAC associated with Ringer lactate or hypertonic saline in the inflammatory response. Bacterial translocation on experimental IO and ischemia were also contemplated.

Our findings show that NAC associated with RL promote a decrease in the inflammatory process in the kidneys and lungs in rats, followed by IO and ischemia. When associated with hypertonic saline, NAC was also able to reduce the degree of histological lesion in the lungs.
